# The iterative bisection procedure: a useful tool for determining parameter values in data-generating processes in Monte Carlo simulations

**DOI:** 10.1186/s12874-023-01836-5

**Published:** 2023-02-17

**Authors:** Peter C. Austin

**Affiliations:** 1 ICES, 2075 Bayview Avenue, Toronto, ON G106M4N 3M5 Canada; 2 Institute of Health Policy, Management and Evaluation, University of Toronto, Toronto, ON Canada; 3 Sunnybrook Research Institute, Toronto, ON Canada

**Keywords:** Data-generating process, Simulations, Monte Carlo simulations

## Abstract

**Background:**

Data-generating processes are key to the design of Monte Carlo simulations. It is important for investigators to be able to simulate data with specific characteristics.

**Methods:**

We described an iterative bisection procedure that can be used to determine the numeric values of parameters of a data-generating process to produce simulated samples with specified characteristics. We illustrated the application of the procedure in four different scenarios: (i) simulating binary outcome data from a logistic model such that the prevalence of the outcome is equal to a specified value; (ii) simulating binary outcome data from a logistic model based on treatment status and baseline covariates so that the simulated outcomes have a specified treatment relative risk; (iii) simulating binary outcome data from a logistic model so that the model c-statistic has a specified value; (iv) simulating time-to-event outcome data from a Cox proportional hazards model so that treatment induces a specified marginal or population-average hazard ratio.

**Results:**

In each of the four scenarios the bisection procedure converged rapidly and identified parameter values that resulted in the simulated data having the desired characteristics.

**Conclusion:**

An iterative bisection procedure can be used to identify numeric values for parameters in data-generating processes to generate data with specified characteristics.

## Introduction

Monte Carlo simulations are a critical tool in modern statistical research [1, 2]. Simulations allow one to investigate the properties of statistical estimators and procedures in settings in which analytic calculations are not feasible. A crucial component of any simulation is a data-generating process that allows the investigator to simulate data with specified characteristics. While a data-generating process can often be quickly constructed, it is more difficult to specify the values of the parameters of the data-generating process to result in the simulated data having specified characteristics.

For instance, given a set of baseline covariates simulated from a multivariate distribution, a logistic model can be used to simulate binary outcomes so as to induce an odds ratio of a specified magnitude for the association between a variable denoting treatment status (treatment vs. control) and the outcome. However, if one wanted to induce a treatment effect with a specific relative risk or risk difference, rather than a specific odds ratio, one would need to identify the given log-odds ratio for treatment that resulted in the desired relative risk or risk difference [3, 4]. This log-odds ratio would depend on the distribution of baseline covariates. Similarly, if one wanted to simulate binary outcome data such that the logistic regression model for the outcome had a specified c-statistic (equivalent to the area under the receiver operating characteristic (ROC) curve), one would need to determine the regression coefficients for the logistic regression model that result in the desired c-statistic.

We describe an iterative bisection procedure that allows researchers to determine the required value of parameters in a data-generating process to result in simulated data with the desired characteristics. We illustrate the iterative bisection procedure by applying it to four different examples. In “Determining the intercept of a logistic regression model so that prevalence of treatment is equal to a specified value when using a logistic regression model to simulate outcomes” section, we apply the iterative bisection procedure to construct a data-generating process for simulating binary outcomes from a multivariable logistic regression model so that the prevalence of the outcome in the population is equal to a specified probability. In “Determining the odds ratio for a binary treatment variable in a logistic regression model to induce a desired treatment risk difference or relative risk in the population” section, we apply the bisection procedure to construct a data-generating process for simulating binary outcomes using a multivariable logistic regression model such that a binary treatment (or exposure) induces a relative risk of a given magnitude. In “Determining the regression coefficients for a logistic regression model so that the model has a specified c-statistic” section, we apply the bisection procedure to construct a data-generating process for simulating binary outcomes from a multivariable logistic regression model with a specified c-statistic. In “Determining the conditional hazard ratio for treatment/exposure in an adjusted Cox regression model to induce a specified marginal hazard ratio” section, we apply the bisection procedure to construct a data-generating process for simulating time-to-event outcomes with a specified marginal hazard ratio for treatment. Finally, we provide a summary in “Discussion” section.

## Determining the intercept of a logistic regression model so that prevalence of treatment is equal to a specified value when using a logistic regression model to simulate outcomes

### Description of method

In this section we consider a setting in which one wants to simulate a binary outcome that is related to a vector of covariates, such that the prevalence of the outcome in the population is equal to a specified value. Let ${\text{P}}^{{{\text{target}}}}$ denote the specified or target prevalence of the outcome in the population.

The first step is to simulate a vector of covariates for each subject in a large super-population, say of size *N* = 1,000,000. The distribution of the baseline covariates can be chosen by the investigator. The application of the bisection procedure is independent of this distributional decision. Assume that we simulate p baseline covariates ($X_{1} , \ldots ,X_{{\text{p}}}$) from a given multivariable distribution.

The second step is to specify a logistic regression model for generating the binary outcomes:

1 $${\text{logit(Pr(Y}}_{i} = 1)) = \beta_{0} + \sum\limits_{j = 1}^{{\text{p}}} {\beta_{j} X_{ij} }$$

The regression coefficients $\beta_{1} , \ldots ,\beta_{{\text{p}}}$ can be chosen by the investigator to reflect the desired relationship between each of the p covariates and the log-odds of the outcome. The prevalence of the outcome in the population is primarily determined by the intercept, $\beta_{0}$. One must determine the value of the intercept that produces the desired prevalence of the outcome. Lower values of $\beta_{0}$ are associated with lower prevalences of the outcome, while higher values of $\beta_{0}$ are associated with higher prevalences of the outcome. For a given value of $\beta_{0}$ we can simulate a binary outcome for each subject in the super-population from a Bernoulli distribution with subject-specific parameter determined by formula (1). Let ${\text{Y}}_{i}^{{\beta_{0} }}$ denote the simulated outcome for the *i*th subject when the intercept for the regression model (1) is set equal to $\beta_{0}$.

The next step is to specify the endpoints of an interval for the parameter of interest; in this case the regression intercept, $\beta_{0}$. Denote this interval by $(\beta_{0}^{{{\text{lower}}}} ,\beta_{0}^{{{\text{upper}}}} )$. The lower endpoint $\beta_{0}^{{{\text{lower}}}}$ is chosen such that $\frac{1}{N}\sum\limits_{i = 1}^{N} {{\text{Y}}_{i}^{{{\beta_{0}{{\text{lower}}}} }} } < {\text{P}}^{{{\text{target}}}}$. In other words, the prevalence of the simulated outcome is less than the target value when using $\beta_{0}^{{{\text{lower}}}}$. Similarly, the upper endpoint $\beta_{0}^{{{\text{upper}}}}$ is chosen such that $\frac{1}{N}\sum\limits_{i = 1}^{N} {{\text{Y}}_{i}^{{{\beta_{0}{{\text{upper}}}} }} } {\text{ > P}}^{{{\text{target}}}}$. In other words, the prevalence of the simulated outcome is greater than the target value when using $\beta_{0}^{{{\text{upper}}}}$. The endpoints can be identified through a grid search or by trial and error.

Once the endpoints of the interval $(\beta_{0}^{{{\text{lower}}}} ,\beta_{0}^{{{\text{upper}}}} )$ have been determined, compute the midpoint of the interval: $\beta_{0}^{{{\text{midpoint}}}} = \frac{{\beta_{0}^{{{\text{lower}}}} + \beta_{0}^{{{\text{upper}}}} }}{2}$ (e.g., if the original endpoints of the interval are ± 10, the original midpoint will be 0). We then use $\beta_{0}^{{{\text{midpoint}}}}$ in formula (1) and simulate a binary outcome for each subject: ${\text{Y}}_{i}^{{{\beta_{0}^{{\text{midpoint}}}} }}$. We then compute the prevalence of the outcome in the super-population: $\frac{1}{N}\sum\limits_{i = 1}^{N} {{\text{Y}}_{i}^{{{\beta_{0}^{{\text{midpoint}}}} }} }$. If $\frac{1}{N}\sum\limits_{i = 1}^{N} {{\text{Y}}_{i}^{{{\beta_{0}^{{\text{midpoint}}}} }} } < {\text{P}}^{{{\text{target}}}}$, the prevalence of the outcome is too low and the intercept of formula (1) has to be increased. If $\frac{1}{N}\sum\limits_{i = 1}^{N} {{\text{Y}}_{i}^{{{\beta_{0}^{{\text{midpoint}}}} }} } {\text{ > P}}^{{{\text{target}}}}$, the prevalence is too high and the intercept of formula (1) has to be decreased.

If $\frac{1}{N}\sum\limits_{i = 1}^{N} {{\text{Y}}_{i}^{{{\beta_{0}^{{\text{midpoint}}}} }} } < {\text{P}}^{{{\text{target}}}}$, then define a new interval: $(\beta_{0}^{{{\text{midpoint}}}} ,\beta_{0}^{{{\text{upper}}}} )$. Conversely, if $\frac{1}{N}\sum\limits_{i = 1}^{N} {{\text{Y}}_{i}^{{{\beta_{0}^{{\text{midpoint}}}} }} } {\text{ > P}}^{{{\text{target}}}}$, then define a new interval $(\beta_{0}^{{{\text{lower}}}} ,\beta_{0}^{{{\text{midpoint}}}} )$. In the first case, the new interval is the upper half of the initial interval, while the in the second case the new interval is the lower half of the initial interval. In either case, the width of the new interval is half the width of the initial interval. We have bisected the initial interval. One then repeats this process iteratively. After K iterations, the width of the resultant interval is $\frac{1}{{2^{K} }}$ of the width of the initial interval. The iterative process can be continued until $\frac{1}{N}\sum\limits_{i = 1}^{N} {{\text{Y}}_{i}^{{{\beta_{0}^{{\text{midpoint}}}} }} }$ is as close to ${\text{P}}^{{{\text{target}}}}$ as desired.

### Application of method

We applied the iterative bisection procedure to simulate data for a sample of size *N* = 1,000,000. We simulated 10 baseline covariates. The first 5 from independent standard normal distributions, and the last five from independent Bernoulli distributions with parameter 0.5. The regression coefficients (equivalent to log-odds ratios) for the 10 covariates were set to $$\begin{gathered} \beta_{1} = \log (1.25),\;\beta_{2} = \log (1.5),\;\beta_{3} = \log (1.75),\;\beta_{4} = \log (2),\;\beta_{5} = \log (2.5),\; \hfill \\ \beta_{6} = \log (1.25),\;\beta_{7} = \log (1.5),\;\beta_{8} = \log (1.75),\;\beta_{9} = \log (2),\;\beta_{10} = \log (2.5). \hfill \\ \end{gathered}$$.

Our objective was to simulate data such that the prevalence of the outcome was 0.10 (10%). The initial interval for $(\beta_{0}^{{{\text{lower}}}} ,\beta_{0}^{{{\text{upper}}}} )$ was set to (-10,10). R code to implement the bisection procedure is provided at the author’s GitHub account [https://github.com/peter-austin/BMC_MRM-bisection-procedures-for-Monte-Carlo-simulations]. The estimates of $\beta_{0}^{{{\text{midpoint}}}} {\text{ and }}\frac{1}{N}\sum\limits_{i = 1}^{N} {{\text{Y}}_{i}^{{{\beta_{0}^{{\text{midpoint}}}} }} }$ at each iteration are reported in Table 1. After 14 iterations of the procedure, an intercept equal to -4.368896 resulted in the generation of outcomes such that the prevalence of the outcome was 0.099923.

**Table 1:** Bisection procedure to determine intercept of a logistic regression model to produce an outcome with a given prevalence (target prevalence: 0.10)

Iteration	Target outcome prevalence	$\beta_{0}^{{{\text{midpoint}}}}$	Empirical outcome prevalence
1	0.1	0	0.729943
2	0.1	-5	0.062318
3	0.1	-2.5	0.313826
4	0.1	-3.75	0.153365
5	0.1	-4.375	0.099513
6	0.1	-4.0625	0.124133
7	0.1	-4.21875	0.111236
8	0.1	-4.29688	0.105887
9	0.1	-4.33594	0.102487
10	0.1	-4.35547	0.100863
11	0.1	-4.36523	0.100523
12	0.1	-4.37012	0.099575
13	0.1	-4.36768	0.100366
14	0.1	-4.3689	0.099923

## Determining the odds ratio for a binary treatment variable in a logistic regression model to induce a desired treatment risk difference or relative risk in the population

### Description of method

The logistic regression model is commonly-used in biomedical and epidemiological research for assessing the association between a binary outcome and a set of covariates [5]. When using a logistic regression model, the odds ratio is the resultant measure of association. The odds ratio denotes the relative increase in the odds of the binary outcome associated with a one unit increase in the given covariate. Other measures of effect for binary outcomes include: the risk difference, the relative risk, and the number needed to treat, where the latter is the reciprocal of the risk difference. Several clinical commentators have suggested that these latter three measures of effect are preferable to the odds ratio for clinical decision making [6–9].

To study the performance of statistical methods for estimating risk differences or relative risks, one requires a data-generating process that can simulate data with a given risk difference or relative risk [3, 4]. We assume that our data-generating process for simulating outcomes is a modification of the one described above. We modify the logistic regression model as follows:

2 $${\text{logit(Pr(Y}}_{i} = 1)) = \beta_{0} + \gamma Z_{i} + \sum\limits_{j = 1}^{{\text{p}}} {\beta_{j} X_{ij} }$$

The model has been modified by including a binary treatment variable (Z = 1 treated; Z = 0 control) with an associated log-odds ratio of $\gamma$. Thus, treatment is associated with an increase of $\gamma$ in the log-odds of the outcome. Let ${\text{RR}}^{{{\text{target}}}}$ denote the target treatment relative risk in the population.

The first step is to simulate baseline covariates $X_{1} , \ldots ,X_{{\text{p}}}$ from a chosen distribution. One can then simulate treatment status using methods described in “Determining the intercept of a logistic regression model so that prevalence of treatment is equal to a specified value when using a logistic regression model to simulate outcomes” section, so that receipt of treatment has a specified association with each of the baseline covariates and so that the prevalence of treatment in the population is equal to the specified value.

The second step is to set the regression coefficients associated with the baseline covariates in formula (2) to the desired quantities. One can use the methods described in “Determining the intercept of a logistic regression model so that prevalence of treatment is equal to a specified value when using a logistic regression model to simulate outcomes” section to determine the intercept ($\beta_{0}$) of formula (2) so that the prevalence of the outcome in the population *if no one were treated* is equal to a specified value.

We introduce the potential outcomes framework, as this facilitates identifying the appropriate value of $\gamma$ [10]. Given a binary treatment Z, let Y(1) and Y(0) denote a subject’s outcomes under treatment (Z = 1) and control (Z = 0) if received under identical circumstances. The average treatment effect (ATE) is defined as E[Y(1) – Y(0)]. The marginal value of the relative risk is defined as E[Y(1)]/E[Y(0)].

The population relative risk due to treatment is determined by the log-odds ratio for treatment, $\gamma$. One must determine the value of $\gamma$ that results in the desired relative risk. As the value of $\gamma$ increases, the relative risk increases. Lower values of $\gamma$ are associated with lower relative risks, while higher values of $\gamma$ are associated with higher relative risks. For a given value of $\gamma$ we can simulate the two potential outcomes for each subject using formula (2). First, we set Z = 0 (control) for all subjects in the super-population and simulate a binary outcome for each subject in the super-population from a Bernoulli distribution with subject-specific parameter determined by formula (2). Let ${\text{Y(0)}}_{i}^{\gamma }$ denote the simulated outcome under control for the *i*th subject when the log-odds ratio for treatment in regression model (2) is set equal to $\gamma$. Second, we set Z = 1 (treated) for all subjects in the super-population and simulate a binary outcome for each subject in the super-population from a Bernoulli distribution with subject-specific parameter determined by formula (2). Let ${\text{Y(1)}}_{i}^{\gamma }$ denote the simulated outcome under treatment for the *i*th subject when log-odds ratio for treatment in regression model (2) is set equal to $\gamma$. The population relative risk when the log-odds ratio for treatment is set to $\gamma$ is equal to ${\text{E[Y(1)}}_{i}^{\gamma } {\text{]/Y[(0)}}_{i}^{\gamma } ] = \frac{{\frac{1}{N}\sum\limits_{i = 1}^{N} {{\text{[Y(1)}}_{i}^{\gamma } ]} }}{{\frac{1}{N}\sum\limits_{i = 1}^{N} {{\text{[Y(0)}}_{i}^{\gamma } ]} }}$.

The next step is to specify the endpoints of an interval for the log-odds ratio for treatment, $\gamma$. Denote this interval by $(\gamma_{{}}^{{{\text{lower}}}} ,\gamma_{{}}^{{{\text{upper}}}} )$. The lower endpoint $\gamma^{{{\text{lower}}}}$ is chosen such that $\frac{{\frac{1}{N}\sum\limits_{i = 1}^{N} {{\text{[Y(1)}}_{i}^{\gamma } ]} }}{{\frac{1}{N}\sum\limits_{i = 1}^{N} {{\text{[Y(0)}}_{i}^{\gamma } ]} }} < {\text{RR}}^{{{\text{target}}}}$. Similarly, the upper endpoint $\gamma^{{{\text{upper}}}}$ is chosen such that $\frac{{\frac{1}{N}\sum\limits_{i = 1}^{N} {{\text{[Y(1)}}_{i}^{\gamma } ]} }}{{\frac{1}{N}\sum\limits_{i = 1}^{N} {{\text{[Y(0)}}_{i}^{\gamma } ]} }}{\text{ > RR}}^{{{\text{target}}}}$. The endpoints can be identified through a grid search or by trial and error.

Once the endpoints of the interval $(\gamma_{{}}^{{{\text{lower}}}} ,\gamma_{{}}^{{{\text{upper}}}} )$ have been determined, compute the midpoint of the interval: $\gamma^{{{\text{midpoint}}}} = \frac{{\gamma^{{{\text{lower}}}} + \gamma^{{{\text{upper}}}} }}{2}$. We then use $\gamma^{{{\text{midpoint}}}}$ in formula (2) and simulate the two potential outcomes under treatment and control for each subject: ${\text{Y(1)}}_{i}^{{\gamma^{{{\text{midpoint}}}} }}$ and ${\text{Y(0)}}_{i}^{{\gamma^{{{\text{midpoint}}}} }}$. We then compute the treatment relative risk in the super-population: $\frac{{\frac{1}{N}\sum\limits_{i = 1}^{N} {{\text{[Y(1)}}_{i}^{{\gamma^{{{\text{midpoint}}}} }} ]} }}{{\frac{1}{N}\sum\limits_{i = 1}^{N} {{\text{[Y(0)}}_{i}^{{\gamma^{{{\text{midpoint}}}} }} ]} }}$. If $\frac{{\frac{1}{N}\sum\limits_{i = 1}^{N} {{\text{[Y(1)}}_{i}^{{\gamma^{{{\text{midpoint}}}} }} ]} }}{{\frac{1}{N}\sum\limits_{i = 1}^{N} {{\text{[Y(0)}}_{i}^{{\gamma^{{{\text{midpoint}}}} }} ]} }} < {\text{RR}}^{{{\text{target}}}}$, the relative risk is too low and $\gamma$ in formula (2) has to be increased. If $\frac{{\frac{1}{N}\sum\limits_{i = 1}^{N} {{\text{[Y(1)}}_{i}^{{\gamma^{{{\text{midpoint}}}} }} ]} }}{{\frac{1}{N}\sum\limits_{i = 1}^{N} {{\text{[Y(0)}}_{i}^{{\gamma^{{{\text{midpoint}}}} }} ]} }}{\text{ > RR}}^{{{\text{target}}}}$, the relative risk is too large and $\gamma$ in formula (2) has to be decreased.

If $\frac{{\frac{1}{N}\sum\limits_{i = 1}^{N} {{\text{[Y(1)}}_{i}^{{\gamma^{{{\text{midpoint}}}} }} ]} }}{{\frac{1}{N}\sum\limits_{i = 1}^{N} {{\text{[Y(0)}}_{i}^{{\gamma^{{{\text{midpoint}}}} }} ]} }} < {\text{RR}}^{{{\text{target}}}}$, then define a new interval: $(\gamma_{{}}^{{{\text{midpoint}}}} ,\gamma_{{}}^{{{\text{upper}}}} )$. Conversely, if $\frac{{\frac{1}{N}\sum\limits_{i = 1}^{N} {{\text{[Y(1)}}_{i}^{{\gamma^{{{\text{midpoint}}}} }} ]} }}{{\frac{1}{N}\sum\limits_{i = 1}^{N} {{\text{[Y(0)}}_{i}^{{\gamma^{{{\text{midpoint}}}} }} ]} }}{\text{ > RR}}^{{{\text{target}}}}$, then define a new interval $(\gamma_{{}}^{{{\text{lower}}}} ,\gamma_{{}}^{{{\text{midpoint}}}} )$. In the first case, the new interval is the upper half of the initial interval, while the in the second case the new interval is the lower half of the initial interval. In either case, the width of the new interval is half the width of the initial interval. We have bisected the initial interval. One then repeats this process iteratively until $\frac{{\frac{1}{N}\sum\limits_{i = 1}^{N} {{\text{[Y(1)}}_{i}^{{\gamma^{{{\text{midpoint}}}} }} ]} }}{{\frac{1}{N}\sum\limits_{i = 1}^{N} {{\text{[Y(0)}}_{i}^{{\gamma^{{{\text{midpoint}}}} }} ]} }} \,$ is as close to ${\text{RR}}^{{{\text{target}}}}$ as desired.

The above procedure allows one to determine the value of $\gamma$ necessary to induce a given treatment relative. The procedure can be modified to induce a given treatment risk difference. To do so, all occurrences of $\frac{{\frac{1}{N}\sum\limits_{i = 1}^{N} {{\text{Y(1)}}_{i}^{\gamma } } }}{{\frac{1}{N}\sum\limits_{i = 1}^{N} {{\text{Y(0)}}_{i}^{\gamma } } }}$ are replaced by $\frac{1}{N}\sum\limits_{i = 1}^{N} {{\text{[Y(1)}}_{i}^{\gamma } {\text{ - Y(0)}}_{i}^{\gamma } ]}$.

### Application of method

We applied the iterative bisection procedure to simulate data for a sample of size *N* = 1,000,000. We simulated 10 baseline covariates as in “Description of method” of “Determining the intercept of a logistic regression model so that prevalence of treatment is equal to a specified value when using a logistic regression model to simulate outcomes” section. We first simulated a binary treatment status variable using formula (1) with the 10 regression coefficients for the baseline covariates in the treatment-selection model set to log(1.1), log(2), log(3), log(1.5), log(1.5), log(1.1), log(2), log(3), log(1.5), and log(1.5). We used the bisection process described in Determining the intercept of a logistic regression model so that prevalence of treatment is equal to a specified value when using a logistic regression model to simulate outcomes” section to determine the intercept for the treatment selection model such that the prevalence of treatment in the population was 0.2. The resultant intercept was -3.31749.

The regression coefficients (equivalent to log-odds ratios) for the 10 covariates in the outcome model (formula (2)) were set to $$\begin{gathered} \beta_{1} = \log (1.25),\;\beta_{2} = \log (1.5),\;\beta_{3} = \log (1.75),\;\beta_{4} = \log (2),\;\beta_{5} = \log (2.5),\;\beta_{6} = \log (1.25),\;\beta_{7} = \log (1.5),\; \hfill \\ \beta_{8} = \log (1.75),\;\beta_{9} = \log (2),\;\beta_{10} = \log (2.5). \hfill \\ \end{gathered}$$

We set the value of $\beta_{0}$ to that determined in the first “Description of method” of “Determining the intercept of a logistic regression model so that prevalence of treatment is equal to a specified value when using a logistic regression model to simulate outcomes” (i.e., the subsection in the Determining the intercept of a logistic regression model so that prevalence of treatment is equal to a specified value when using a logistic regression model to simulate outcomes section) section ($\beta_{0}$ = -4.367676) so that the prevalence of outcome was 0.10 (10%) if no subjects were treated.

Our objective was to simulate data such that the treatment relative risk is 0.80. The initial interval for $\gamma$ was set to (-10,10). R code to implement the bisection procedure is provided at the author’s GitHub account [https://github.com/peter-austin/BMC_MRM-bisection-procedures-for-Monte-Carlo-simulations]. The estimates of $\gamma_{{}}^{{{\text{midpoint}}}} {\text{ and }}\frac{{\frac{1}{N}\sum\limits_{i = 1}^{N} {{\text{Y(1)}}_{i}^{\gamma } } }}{{\frac{1}{N}\sum\limits_{i = 1}^{N} {{\text{Y(0)}}_{i}^{\gamma } } }}$ at each iteration are reported in Table 2. After 16 iterations, the procedure identified that a treatment log-odds ratio of -0.2999878 (equivalent to an odds ratio of 0.741) resulted in a relative risk of 0.8000121.

**Table 2:** Bisection procedure to determine log-odds ratio for treatment in a logistic regression model to produce a binary outcome with a given relative risk (target relative risk: 0.80)

Iteration	Target relative risk	$\gamma_{{}}^{{{\text{midpoint}}}}$	Empirical relative risk
1	0.8	0	1
2	0.8	-5	0.010792
3	0.8	-2.5	0.12165
4	0.8	-1.25	0.371719
5	0.8	-0.625	0.621492
6	0.8	-0.3125	0.792433
7	0.8	-0.15625	0.891377
8	0.8	-0.23438	0.840727
9	0.8	-0.27344	0.81629
10	0.8	-0.29297	0.804289
11	0.8	-0.30273	0.798343
12	0.8	-0.29785	0.801312
13	0.8	-0.30029	0.799827
14	0.8	-0.29907	0.800569
15	0.8	-0.29968	0.800198
16	0.8	-0.29999	0.800012

## Determining the regression coefficients for a logistic regression model so that the model has a specified c-statistic

### Description of method

The c-statistic (equivalent to the area under the receiver operating characteristic (ROC) curve, which is sometimes abbreviated as the AUC) is a measure of discrimination used to assess the predictive performance of logistic regression models [11, 12]. In this section we describe how to specify the coefficients of a logistic regression model to simulate outcomes such that the underlying logistic regression model has a specified c-statistic. In doing so, we make use of the fact that the c-statistic of a univariate logistic regression model is a function of the variance of the covariate and the log-odds ratio for that covariate [13]. Let ${\text{AUC}}^{{{\text{target}}}}$ denote the target c-statistic.

We modify the logistic regression model described in formula (1):

3 $${\text{logit(Pr(Y}}_{i} = 1)) = \beta_{0} + \sigma \sum\limits_{j = 1}^{{\text{p}}} {\beta_{j} X_{ij} }$$

The regression coefficients $\beta_{1} , \ldots ,\beta_{{\text{p}}}$ can be chosen by the investigator to reflect the desired relationship between each of the covariates and the log-odds of the outcome. By introducing the scalar $\sigma$, we are modifying each log-odds ratio, but doing so in such a way that the ratio of any two log-odds ratios remains constant after modification. We need to identify the value of $\sigma$ required to induce the desired c-statistic. Larger values of $\sigma$ are associated with larger values of the c-statistic, while lower values of $\sigma$ are associated with smaller values of the c-statistic.

The first step is to simulate a vector of covariates for each subject in a large super-population, say of size *N* = 1,000,000. The distribution of the baseline covariates can be chosen by the investigator. The application of the bisection procedure is independent of this distributional decision.

For a given value of $\sigma$ we can simulate a binary outcome for each subject in the super-population from a Bernoulli distribution with subject-specific parameter determined by formula (3). Let ${\text{Y}}_{i}^{\sigma }$ denote the simulated outcome for the *i*th subject when $\sigma$ is a constant scalar as shown in formula (3).

The next step is to specify the endpoints of an interval for $\sigma$. Denote this interval by $(\sigma^{{{\text{lower}}}} ,\sigma^{{{\text{upper}}}} )$. The lower endpoint $\sigma^{{{\text{lower}}}}$ is chosen such that when binary outcomes are simulated using a Bernoulli distribution with subject-specific parameter determined using formula (3), the c-statistic of the logistic regression model fit to the simulated data has a c-statistic that is less than ${\text{AUC}}^{{{\text{target}}}}$. Similarly, the upper endpoint $\sigma^{{{\text{upper}}}}$ is chosen that when binary outcomes are simulated using a Bernoulli distribution with subject-specific parameter determined using formula (3), the c-statistic of the logistic regression model fit to the simulated data has a c-statistic that is greater than ${\text{AUC}}^{{{\text{target}}}}$. The endpoints can be identified through a grid search or by trial and error.

Once the endpoints of the interval $(\sigma^{{{\text{lower}}}} ,\sigma^{{{\text{upper}}}} )$ have been determined, compute the midpoint of the interval: $\sigma^{{{\text{midpoint}}}} = \frac{{\sigma^{{{\text{lower}}}} + \sigma^{{{\text{upper}}}} }}{2}$. We then use $\sigma^{{{\text{midpoint}}}}$ in formula (3) and simulate a binary outcome for each subject: ${\text{Y}}_{i}^{{\sigma^{{{\text{midpoint}}}} }}$. We fit a logistic regression model in the simulated sample and determine its c-statistic, which we refer to as ${\text{AUC}}^{{\sigma^{{{\text{midpoint}}}} }}$. If ${\text{AUC}}^{{\sigma^{{{\text{midpoint}}}} }} < {\text{AUC}}^{{{\text{target}}}}$, the c-statistic is too low and $\sigma$ has to be increased. If ${\text{AUC}}^{{\sigma^{{{\text{midpoint}}}} }} {\text{ > AUC}}^{{{\text{target}}}}$, the c-statistic is too large and $\sigma^{{{\text{midpoint}}}}$ has to be decreased.

If ${\text{AUC}}^{{\sigma^{{{\text{midpoint}}}} }} < {\text{AUC}}^{{{\text{target}}}}$, then define a new interval: $(\sigma^{{{\text{midpoint}}}} ,\sigma^{{{\text{upper}}}} )$. Conversely, if ${\text{AUC}}^{{\sigma^{{{\text{midpoint}}}} }} {\text{ > AUC}}^{{{\text{target}}}}$, then define a new interval $(\sigma^{{{\text{lower}}}} ,\sigma^{{{\text{midpoint}}}} )$. One then repeats this process iteratively until ${\text{AUC}}^{{\sigma^{{{\text{midpoint}}}} }}$ is as close to ${\text{AUC}}^{{{\text{target}}}}$ as desired.

### Application of method

We applied the iterative bisection procedure to simulate data for a sample of size *N* = 1,000,000. We simulated 10 baseline covariates as in “Description of method” of “Determining the intercept of a logistic regression model so that prevalence of treatment is equal to a specified value when using a logistic regression model to simulate outcomes” section. As above, the regression coefficients (equivalent to log-odds ratios) for the 10 covariates in the outcome model (formula (3)) were set to $$\begin{gathered} \beta_{1} = \log (1.25),\;\beta_{2} = \log (1.5),\;\beta_{3} = \log (1.75),\;\beta_{4} = \log (2),\;\beta_{5} = \log (2.5),\;\beta_{6} = \log (1.25),\;\beta_{7} = \log (1.5),\; \hfill \\ \beta_{8} = \log (1.75),\;\beta_{9} = \log (2),\;\beta_{10} = \log (2.5). \hfill \\ \end{gathered}$$

We set the value of $\beta_{0}$ to that determined in the first “Description of method” of “Determining the intercept of a logistic regression model so that prevalence of treatment is equal to a specified value when using a logistic regression model to simulate outcomes” section ($\beta_{0}$ = -4.368896).

Our objective was to simulate binary outcomes such that c-statistic of the logistic regression model was 0.8. The initial interval for $\sigma$ was set to (0,10). R code to implement the bisection procedure is provided at the author’s GitHub account [https://github.com/peter-austin/BMC_MRM-bisection-procedures-for-Monte-Carlo-simulations. The values of $\sigma_{{}}^{{{\text{midpoint}}}} ,{\text{ and AUC}}^{{\sigma^{{{\text{midpoint}}}} }}$ at each iteration are reported in Table 3. After 11 iterations of the bisection procedure, $\sigma = 0.8349609$ resulted in an empirical c-statistic of 0.8006215.

**Table 3:** Bisection procedure to determine $\sigma$ for multiplying the coefficients of a logistic regression model to produce a binary outcome such that the outcomes model has a given c-statistic (target c-statistic: 0.80)

Iteration	Target c-statistic	$\sigma_{{}}^{{{\text{midpoint}}}}$	Empirical c-statistic ${\text{(AUC}}^{{\sigma^{{{\text{midpoint}}}} }} )$
1	0.8	5	0.983782
2	0.8	2.5	0.944541
3	0.8	1.25	0.868001
4	0.8	0.625	0.742962
5	0.8	0.9375	0.821941
6	0.8	0.78125	0.788305
7	0.8	0.859375	0.805549
8	0.8	0.820313	0.79756
9	0.8	0.839844	0.801353
10	0.8	0.830078	0.798494
11	0.8	0.834961	0.800622

## Determining the conditional hazard ratio for treatment/exposure in an adjusted Cox regression model to induce a specified marginal hazard ratio

### Description of method

The Cox proportional hazard regression is frequently used in biomedical and epidemiological research [14]. When fitting a multivariable Cox regression model, the regression coefficients, when exponentiated, are interpretated as conditional (or adjusted) hazard ratios. For a given covariate, the conditional hazard ratio compares the relative difference in the hazard of the outcome between two subjects for whom the covariate in question differs by one unit and for whom all the other covariates are identical. In contrast to the conditional (or adjusted) hazard ratio is the marginal (or population-average) hazard ratio. The marginal hazard ratio denotes the relative difference in the hazard function between two populations, for whom the covariate in question differs by one unit, and all other covariates are identical between populations. Due to the phenomenon known as the non-collapsibility of the hazard ratio, marginal and conditional hazard ratios do not coincide (unless one is null) [15].

Bender and colleagues have described data-generating processes for time-to-event outcomes based on an underlying hazard regression model [16]. This data-generating process simulates time-to-event outcomes with specified conditional hazard ratios. One can use the bisection approach with Bender’s data-generating process to induce data with a desired marginal effect for a given covariate.

Assume that we simulate p baseline covariates ($X_{1} , \ldots ,X_{{\text{p}}}$) from a given multivariable distribution. Furthermore, assume that we have the following Cox proportional hazards model:

4 $${\text{log(h}}_{i} (t)) = {\text{log(h}}_{0} (t))+ \gamma Z_{i} + \sum\limits_{j = 1}^{p} {\beta_{j} X_{ij} }$$

where $h_{i} (t)$ denotes the hazard function for the *i*th subject, h_0_(*t*) denotes the baseline hazard function, and where Z denotes a binary treatment variable (Z = 1 treated; Z = 0 control) with an associated conditional log-hazard ratio of $\gamma$. Thus, treatment is associated with an increase of $\gamma$ in the log-hazard of the outcome. Let ${\text{HR}}^{{{\text{target}}}}$ denote the target marginal hazard ratio in the population.

The first step is to simulate baseline covariates $X_{1} , \ldots ,X_{{\text{p}}}$ from a chosen distribution. One can then simulate treatment status (Z) using methods described above, so that receipt of treatment has a specified association with each of the baseline covariates and the prevalence of treatment in the population is equal to the specified value.

The second step is to set the regression coefficients associated with the baseline covariates in formula (4) to the desired quantities.

As above, we use the potential outcomes framework. The marginal hazard ratio due to treatment is determined by the log-hazard ratio for treatment, $\gamma$. One must determine the value of $\gamma$ that results in the desired marginal hazard ratio. For a given value of $\gamma$ we can use Bender’s approach to simulate the two potential outcomes for each subject using formula (4). First, we set Z = 0 (control) for all subjects in the super-population and simulate a time-to-event outcome for each subject in the super-population. Let ${\text{T(0)}}_{i}^{\gamma }$ denote the simulated outcome under control for the *i*th subject when the log-hazard ratio for treatment in regression model (4) is set to $\gamma$. Second, we set Z = 1 (treated) for all subjects in the super-population and simulate a time-to-event outcome for each subject in the super-population. Let ${\text{T(1)}}_{i}^{\gamma }$ denote the simulated outcome under treatment for the *i*th subject when the log-hazard ratio for treatment in regression model (4) is set to $\gamma$. One then creates a large super-population by concatenating the two simulated datasets (one under control and one under treatment). Using a univariate Cox proportional hazards model, one then regresses the hazard of the outcome on the variable denoting treatment status. The resultant hazard ratio is an estimate of the marginal hazard ratio.

The next step is to specify the endpoints of an interval for the log-hazard ratio for treatment, $\gamma$. Denote this interval by $(\gamma_{{}}^{{{\text{lower}}}} ,\gamma_{{}}^{{{\text{upper}}}} )$. The lower endpoint $\gamma^{{{\text{lower}}}}$ is chosen such that the estimated marginal hazard ratio is less than ${\text{HR}}^{{{\text{target}}}}$. Similarly, the upper endpoint $\gamma^{{{\text{upper}}}}$ is chosen such that the estimated marginal hazard ratio is greater than ${\text{HR}}^{{{\text{target}}}}$. The endpoints can be identified through a grid search or by trial and error. 

Once the endpoints of the interval $(\gamma_{{}}^{{{\text{lower}}}} ,\gamma_{{}}^{{{\text{upper}}}} )$ have been determined, compute the midpoint of the interval: $\gamma^{{{\text{midpoint}}}} = \frac{{\gamma^{{{\text{lower}}}} + \gamma^{{{\text{upper}}}} }}{2}$. We then use $\gamma^{{{\text{midpoint}}}}$ in formula (4) and simulate the two potential outcomes under treatment and control for each subject: ${\text{T(1)}}_{i}^{{\gamma^{{{\text{midpoint}}}} }}$ and ${\text{T(0)}}_{i}^{{\gamma^{{{\text{midpoint}}}} }}$. We then compute the marginal treatment when the dataset consisting of both potential outcomes for all subjects is used to regress the hazard of the outcome on treatment status. If the estimated marginal hazard ratio is less than ${\text{HR}}^{{{\text{target}}}}$, the hazard ratio is too low and $\gamma$ in formula (4) must be increased. If the estimated marginal hazard ratio is greater than ${\text{HR}}^{{{\text{target}}}}$, the hazard ratio is too large and $\gamma$ in formula (4) must be decreased.

If the estimated marginal hazard ratio is less than ${\text{HR}}^{{{\text{target}}}}$, then define a new interval: $(\gamma_{{}}^{{{\text{midpoint}}}} ,\gamma_{{}}^{{{\text{upper}}}} )$. Conversely, if the estimated marginal hazard ratio is greater than ${\text{HR}}^{{{\text{target}}}}$, then define a new interval $(\gamma_{{}}^{{{\text{lower}}}} ,\gamma_{{}}^{{{\text{midpoint}}}} )$. In the first case, the new interval is the upper half of the initial interval, while the in the second case the new interval is the lower half of the initial interval. In either case, the width of the new interval is half the width of the initial interval. We have bisected the initial interval. One then repeats this process iteratively until the estimated marginal hazard ratio is as close to the target marginal hazard ratio, ${\text{HR}}^{{{\text{target}}}}$, as desired.

### Application of method

We applied the iterative bisection procedure to simulate data for a sample of size *N* = 1,000,000. We simulated 10 baseline covariates as in “Description of method” of “Determining the intercept of a logistic regression model so that prevalence of treatment is equal to a specified value when using a logistic regression model to simulate outcomes” section. We used the regression coefficients for the treatment-selection model described in “Description of method” of “Determining the odds ratio for a binary treatment variable in a logistic regression model to induce a desired treatment risk difference or relative risk in the population” section, with the same intercept as determined in “Description of method” of “Determining the odds ratio for a binary treatment variable in a logistic regression model to induce a desired treatment risk difference or relative risk in the population” section, so that the prevalence of treatment was 0.20.

The regression coefficients for the Cox regression model described in formula (4) were set to $$\begin{gathered} \beta_{1} = \log (1.25),\;\beta_{2} = \log (1.5),\;\beta_{3} = \log (1.75),\;\beta_{4} = \log (2),\;\beta_{5} = \log (2.5),\;\beta_{6} = \log (1.25),\;\beta_{7} = \log (1.5),\; \hfill \\ \beta_{8} = \log (1.75),\;\beta_{9} = \log (2),\;\beta_{10} = \log (2.5). \hfill \\ \end{gathered}$$

Our objective was to simulate data such that the marginal hazard ratio for treatment was 0.80. The initial interval for $\gamma$ was set to (-10,10). R code to implement the bisection procedure is provided at the author’s GitHub account [https://github.com/peter-austin/BMC_MRM-bisection-procedures-for-Monte-Carlo-simulations]. The estimates of $\gamma_{{}}^{{{\text{midpoint}}}} {\text{ and HR}}_{{{\text{marginal}}}}^{{\gamma_{{}}^{{{\text{midpoint}}}} }}$ at each iteration are reported in Table 4. After 12 iterations of the bisection procedure, a conditional log-hazard ratio for treatment equal to 0.6298828 resulted in simulated outcomes with a marginal hazard ratio of 0.7991514.

**Table 4:** Bisection procedure to determine conditional log-hazard ratio for treatment in a Cox proportional hazards model to produce a time-to-event outcome with a given marginal hazard ratio for treatment (target marginal hazard ratio for treatment: 0.80)

Iteration	Target marginal hazard ratio	$\gamma_{{}}^{{{\text{midpoint}}}} \,$	Empirical marginal hazard ratio ${\text{(HR}}_{{{\text{marginal}}}}^{{\gamma_{{}}^{{{\text{midpoint}}}} }} )$
1	0.8	0	0.000354
2	0.8	5	2.203321
3	0.8	2.5	1.561186
4	0.8	1.25	1.114086
5	0.8	0.625	0.796174
6	0.8	0.9375	0.969266
7	0.8	0.78125	0.887425
8	0.8	0.703125	0.843299
9	0.8	0.664063	0.820148
10	0.8	0.644531	0.808168
11	0.8	0.634766	0.802396
12	0.8	0.629883	0.799151

## Discussion

We illustrated the application of an iterative bisection procedure that allows investigators to select the numeric values of parameters in a data-generating process to produce simulated datasets with specified characteristics. This will facilitate designing data-generating processes that produce simulated datasets that are tailored to the investigators’ specifications.

We illustrated the use of the bisection procedure when there is one characteristic that requires specification (e.g., the prevalence of the outcome or the c-statistic of a logistic regression model). The procedure can be modified to simulate data such that two characteristics are fixed at specified values (e.g., both the prevalence of the outcome and the c-statistic of the logistic regression model). To do so, one would apply the procedure sequentially and then iteratively repeat the sequential process until both characteristics are close to the target values. It is necessary to repeat the process iteratively as modifying the parameter values during the second application of the procedure (e.g., for the c-statistic of the regression model) may modify the value of the first characteristic (e.g., the prevalence of the outcome).

The bisection procedure has been successfully used in previous studies that used simulations to: assess the ability to rank hospitals by their performance on composite indicators [17], describe a data-generating process for data with a specified marginal odds ratio [18], describe a data-generating process for data with a specified risk difference or number needed to treat [19], to determine the rate parameter for an exponential censoring distribution so as to induce the desired proportion of censoring [20], in a study of the performance of double propensity score adjustment [21], in a study on the performance of the generalized propensity score for estimating the effect of quantitative exposures on time-to-event outcomes [22], to assess the performance of propensity score methods for estimating marginal hazard ratios [23], in a study of the use of the bootstrap with propensity score matching [24], in a comparison of algorithms for matching on the propensity score [25], assess the use of optimal matching with survival outcomes [26], to assess methods of variance estimation when using inverse probability of treatment weighting with survival outcomes [27], to assess the use of propensity score matching in the presence of competing risks [28], to assess the performance of calibration metrics for survival models [29], to assess the performance of variance estimators for survival outcomes when using propensity score matching with replacement [30], to assess the effect of constraints on the matching ratio when using full matching [31], to examine the consequences multiply-imputing missing potential outcomes under control [32], and to examine sample size and power calculations when using inverse probability of treatment weighting [33].

## Conclusion

We have a described an iterative bisection procedure that can be used in designing data-generating processes that produce simulated datasets with specific characteristics.

## Data Availability

No data were used in the study. The software code for simulating artificial datasets is provided on the author’s GitHub account [https://github.com/peter-austin/BMC_MRM-bisection-procedures-for-Monte-Carlo-simulations].

## Abbreviations

AUC: Area under the curve
RR: Relative risk
HR: Hazard ratio
ROC: Receiver operating characteristic
